# The association between maternal and partner experienced racial discrimination and prenatal perceived stress, prenatal and postnatal depression: findings from the growing up in New Zealand cohort study

**DOI:** 10.1186/s12939-016-0443-4

**Published:** 2016-09-22

**Authors:** Laia Bécares, Polly Atatoa-Carr

**Affiliations:** 1 University of Manchester, Humanities Bridgeford Street, G.14, Oxford Road, Manchester, M13 9PL UK; 2 Faculty of Medical and Health Sciences, The University of Auckland, Auckland, 1142 New Zealand

**Keywords:** Racial discrimination, Ethnicity, New Zealand, Prenatal, Postnatal, Depression, Perceived stress

## Abstract

**Background:**

A growing number of studies document the association between maternal experiences of racial discrimination and adverse children’s outcomes, but our understanding of how experiences of racial discrimination are associated with pre- and post-natal maternal mental health, is limited. In addition, existent literature rarely takes into consideration racial discrimination experienced by the partner.

**Methods:**

We analysed data from the Growing Up in New Zealand study to examine the burden of lifetime and past year experiences of racial discrimination on prenatal and postnatal mental health among Māori, Pacific, and Asian women in New Zealand (NZ), and to study the individual and joint contribution of mother’s and partner’s experiences of lifetime and past year racial discrimination to women’s prenatal and postnatal mental health.

**Results:**

Our findings show strong associations between lifetime and past year experiences of ethnically-motivated interpersonal attacks and unfair treatment on mother’s mental health. Māori, Pacific, and Asian women who had experienced unfair treatment by a health professional in their lifetime were 66 % more likely to suffer from postnatal depression, compared to women who did not report these experiences. We found a cumulative effect of lifetime experiences of ethnically-motivated personal attacks on poor maternal mental health if both the mother and the partner had experienced a racist attack.

**Conclusions:**

Experiences of racial discrimination have severe direct consequences for the mother’s mental health. Given the importance of mother’s mental health for the basic human needs of a healthy child, racism and racial discrimination should be addressed.

## Background

Racism, a societal system in which population groups are divided into ‘races,’ with power unequally distributed based on these racial classifications [1], is a well-established determinant of morbidity and mortality which maintains and reproduces ethnic inequities across generations via institutional and interpersonal mechanisms. Institutionally, racism expressed through systemic oppression existent in social institutions ensures that both socioeconomic disadvantage, and its detrimental effect on health [2–5], are continuously transmitted throughout generations. Interpersonally, racism and racial discrimination experienced by mothers before, during, and after pregnancy have been associated with a wide range of adverse children’s outcomes, including low and very low birth weight, preterm birth, higher cortisol reactivity, and cognitive and socioemotional difficulties [6–16]. Stress elicited by chronic exposure to racial discrimination is a key mechanism in the interpersonal transmission of ethnic inequalities in health, not only because of the association between maternal stress during pregnancy and the increased likelihood of preterm birth, low birthweight, developmental delays, and behavioural abnormalities in children [16–20], but also because of the association between stress and the development of mental disorders among mothers.

Despite a growing volume of studies documenting the association between maternal experiences of racial discrimination and adverse children’s outcomes [8, 9, 21–26], the literature on experienced racial discrimination and maternal mental health during pregnancy, or in the perinatal period, is limited. The only four studies that to date have documented these associations have been conducted in individual regions of the US (Michigan, the Boston area, Pittsburgh, and New York City), mostly with African American populations, and have all used lifetime measures of racial discrimination (i.e., measures asking whether respondents have ever experienced racial discrimination) [27–30]. Studies that examine the association between racial discrimination and mental health in the pre- and post-natal periods across other populations, in different national settings, and with experiences of racial discrimination occurring not only at any point in women’s lifetime, but specifically around the conception and perinatal period, are needed in order to better understand the role of racism and racial discrimination in perpetuating intergenerational ethnic inequities in health.

Existent studies on the associations between racial discrimination and children’s outcomes have focused on experiences of racial discrimination reported by the mother. Although the mother is at the centre of the intergenerational transmission of embodied inequity, and her own experiences of racial discrimination may be directly associated with the health and development of her offspring, it is also likely that racial discrimination experienced by those in her proximal social network may lead, through vicarious exposure, to increased levels of stress during pregnancy, and may have detrimental consequences for the foetus. Vicarious exposure to racial discrimination has been found to be harmful to the health of non-pregnant female and male populations [6, 8, 10, 21, 31], and studies of workplace discrimination show an increase in children’s externalising behaviours among children whose fathers have experienced discrimination [32]. However, how vicarious exposure to racial discrimination is associated with women’s mental health during pregnancy is currently unknown, and merits further examination due to its implications for the health of the mother and the child [33].

This study contributes to the literature by exploring, among a diverse sample of pregnant women in New Zealand (NZ), the association between mother’s and partner’s lifetime and past year experiences of racial discrimination, and prenatal and postnatal mental health. New Zealand is an important context in which to study experiences of racial discrimination among pregnant women and their partners, and their association with maternal mental health, because of the longstanding existence of racism and marginalisation that has resulted from historical and ongoing colonisation, and because of the documented stark ethnic inequities in early life child outcomes including preterm birth, small for gestational age, and infant death rates [34–37]. Several studies have shown a persistent detrimental association between experiences of racial discrimination and poor mental and physical health of Māori, the indigenous people of New Zealand, and of migrant and post-migrant communities, including Pacific peoples and Asian ethnic groups [14, 38–45], but to date, no studies have examined direct and vicarious exposure to racial discrimination, and their association with prenatal and postnatal maternal mental health. A recent study on an ethnically diverse group of women from Auckland found that women who reported lifetime experiences of ethnic discrimination had higher evening cortisol and poorer self-rated health in late pregnancy, as compared to women who did not report experiences of racial discrimination [14]. Study results also showed, among a small sample of infants (*N* = 19), higher stress reactivity at 6 weeks of age, suggesting that ethnic discrimination experienced by the mother has biological effects that are transmitted to the next generation [14]. The present study further contributes to the literature by exploring how maternal and partner’s experience of racial discrimination, ever and in a time period close to conception and birth, is associated with prenatal and postnatal maternal mental health. Specifically, the study aims to:

1. Document the prevalence of lifetime and past year experiences of racial discrimination among pregnant women and their partners.
2. Examine the burden of lifetime and past year experiences of racial discrimination on prenatal and postnatal mental health among Māori, Pacific, and Asian women.
3. Examine the individual and joint contribution of mother’s and partner’s experiences of lifetime and past year racial discrimination to women’s prenatal and postnatal mental health.

## Methods

Growing Up in New Zealand (GUiNZ) is a longitudinal study that aims to provide an up-to-date picture of what it is like to be a child growing up in New Zealand in the 21st century [46]. All pregnant women with an estimated delivery date between 25th April 2009 and 25th March 2010, residing within a geographical region defined by the three contiguous District Health Boards (DHBs) of Auckland, Counties Manukau and Waikato, were eligible to participate [46]. GUiNZ recruited 6822 pregnant women, who were comparable to the most recent NZ national birth statistics in relation to maternal age, ethnicity, parity and indicators of socioeconomic position [47]. A total of 4401 of pregnant mother’s partners also enrolled in the study poll. The large majority (99 %) of the partners who were recruited identified themselves as the biological fathers of the cohort children.

Four data collection waves have been carried out with the GUiNZ cohort to date, and this study uses data from the first two interviews. The antenatal interview consisted of a face-to-face Computer Assisted Personal Interview (CAPI) with the pregnant mother (most often in the last trimester of her pregnancy), and with her partner independently, completed in late 2010 [46]. The second face-to-face CAPI with mothers and partners took place when the GUiNZ children were 9 months old [46]. Ninety-four percent of mothers completed the 9 month interview. Ethical approval was obtained from The University of Auckland Human Participants Ethics Committee. Written informed consent was obtained from all participating women, and independently from all participating partners.

### Maternal mental health

We used three measures of maternal mental health: prenatal perceived stress, prenatal depression, and postnatal depression.

Prenatal perceived stress was assessed with the 10-item Perceived Stress Scale (PSS) [48], which asked respondents how often in the last 4 weeks they experienced a range of symptoms of stress, including feeling “unable to control the important things in life,” “nervous and stressed,” and “angered because of things that were outside of your control.” We reversed the scores on four positive items and summed all items (Mean = 14.55, SD = 6.62, Cronbach’s α = 0.85) to create a continuous measure with higher scores representing experiencing more stress.

Prenatal and postnatal depression were measured using the Edinburgh Postnatal Depression Scale (EPDS) [49], a widely used measure of postnatal depression which has also been validated for use during pregnancy [50]. The EPDS consists of 10 questions which ask respondents how they have felt in the past 7 days on items such as “I have felt scared or panicky for no very good reason,” “I have been so unhappy that I have been crying,” and “I have been so unhappy that I have had difficulty sleeping.”

As with the PSS, we reversed scores on positive items and summed all items (Cronbach’s α = 0.85 for both prenatal and postnatal depression). We modelled the EPDS as a dichotomous variable, following the recommended threshold of >12, shown to identify participants likely to be suffering from a depressive illness [49].

#### Racial discrimination

In the antenatal questionnaire, mothers and partners were asked a series of questions regarding lifetime and past year experiences of racial discrimination, covering two broad themes of personal attacks, and unfair treatment. Questions on personal attacks asked respondents whether they had ever felt they were the victim of an ethnically motivated attack (verbal or physical abuse). Response options to this question were: Yes, verbal – within the past 12 months; Yes, verbal – more than 12 months ago; Yes, physical – within the past 12 months; Yes, physical, more than 12 months ago; No. We recoded these responses into four different dummy variables to capture whether participants had experienced any physical abuse in their lifetime, any physical abuse in the past 12 months, any verbal abuse in their lifetime, or any verbal abuse in the past 12 months.

Questions on unfair treatment asked respondents whether they had ever felt they had been treated unfairly because of their ethnicity in New Zealand across a range of domains, including by a health professional; in employment settings; in the housing market; by the police, the justice system, or the corrections department; by the banking system (asking for loans, a mortgage, hire purchase or credit cards); and when attending a place of learning. We used the available response categories of ‘yes, within the past 12 months;’ ‘Yes, more than 12 months ago;’ and ‘No,’ to create twelve dummy variables capturing whether respondents had been treated unfairly in each of these domains, ever or within the past 12 months.

To examine the dose-response relationship between the number of types of racial discrimination and mental health, we summed experiences of personal attacks and unfair treatment, and categorised responses into ‘one experience,’ or ‘two or more experiences.’ We did this separately for lifetime and past year experiences of racial discrimination.

We also created four variables that summarised whether mothers and their partners had experienced any personal attack in their lifetime, any personal attack in the last 12 months, any unfair treatment in their lifetime, or any unfair treatment in the last 12 months, combining responses for each of these variables from the mothers and the partners into ‘only experienced by the mother,’ ‘experienced by both,’ and ‘only experienced by the partner.’

#### Ethnicity

In the antenatal questionnaire, mothers and partners were asked “which ethnic group or groups do you belong to?” Participants were able to provide a description of their ethnic group at a detailed level (corresponding to Statistics New Zealand Level 3/4 hierarchy), with interviewers able to refer to a list of 32 possible answers as well as an open ended ‘Other, please specify’ category. Multiple responses were collected. GUiNZ data available to external researchers consists of Level 1 categorisations, which combine the response categories into European, Māori, Pacific, Asian, and Other ethnic groups. Given the large heterogeneity in the Other category, analyses presented here are restricted to participants of European, Māori, Pacific, and Asian ethnic identity.

Following the use of ethnicity data in other New Zealand studies of racial discrimination and health [39–41], we used two different operationalisations of ethnic grouping, depending on the analyses conducted. To calculate crude prevalence rates we used total response output for the European, Māori, Pacific, and Asian groups, whereby each respondent was counted in each of the ethnic groups that they self-identified with. To examine the association between racial discrimination and maternal mental health, we used prioritisation of ethnicity by assigning each person to a single, mutually exclusive ethnic group. For people with multiple ethnicities, these were prioritised in the following order: Māori, Pacific, Asian, and European. This approach is consistent with the existent literature [39–41].

#### Covariates

We included the following covariates in the analyses: mother’s age, household income, maternal education, mother’s relationship status (with or without partner), and area-level deprivation, assessed using the New Zealand Deprivation Index 2006 [51].

Models examining the association between racial discrimination and maternal mental health among all ethnic minority mothers also adjusted for ethnicity. Models examining the association between racial discrimination and postnatal depression additionally adjusted for prenatal depression (EPDS score in the antenatal interview).

#### Analyses

We first calculated crude prevalence estimates for each of the individual lifetime and past year measures of racial discrimination, and the summary variables, among mothers and their partners of different ethnic groups. In order to understand the association between lifetime and past year experiences of racial discrimination, and the three measures of mental health (prenatal perceived stress, prenatal depression, postnatal depression), we conducted a set of linear and logistic regressions. Because we are interested in the association between racial discrimination and mental health of ethnic minority mothers, this set of analysis was restricted to respondents who self-identified as Māori, Pacific, or Asian (*n* = 3355). Tests for a differential association between measures of racial discrimination and mental health did not provide any evidence that these associations vary by ethnic minority group, so we analysed a pooled sample of women from Māori, Pacific, and Asian ethnic background. Analyses adjusted for the relationship status of the mother, but did not differentiate whether mother’s partners had also completed an interview.

We also used linear and logistic regressions to examine the association between lifetime and past year experiences of racial discrimination among the four categories of maternal and partner experiences of racial discrimination, across the two different time periods, and the three measures of mental health. This set of analysis excluded data from mothers whose partners did not complete an antenatal interview, but did not restrict the sample based on mother’s or partner’s ethnicity, so that mothers and partners of European descent were also included, since they can have a partner of an ethnic minority or indigenous background (*n* = 4118).

We used complete-case analyses of the GUiNZ. The GUiNZ study applies a geographical sampling frame which does not include a complex sampling design with stratification, clustering, or disproportionate selection, and as such, does not require post-hoc weights to be added to the analyses [52, 53]. All analyses were conducted using Stata 13 [54].

## Results

Māori and Pacific Islander were younger and with fewer educational qualifications and lower income. They also lived in the areas with the highest deprivation. In contrast, New Zealand European women tended to be older, and had higher educational qualifications and higher income. Pacific Islander women had the highest rates of prenatal and postnatal depression, and the highest levels of prenatal stress (see Table 1).

**Table 1:** Sociodemographic characteristics of mothers in the Growing Up in New Zealand birth cohort study

	Māori *n* = 1,260	Pacific *n* = 1,029	Asian *n* = 1,051	European *n* = 3,265
	%	%	%	%
Prenatal depression	21.3	28.6	17.2	10.6
Prenatal perceived stress, M (SD)	14.96 (6.9)	15.81 (6.4)	12.85 (6.2)	12.01 (5.9)
Postnatal depression	13.2	19.3	13.1	6.9
Age, M (SD)	27.42 (6.4)	28.26 (6.3)	30.01 (4.7)	31.54 (5.3)
Income				
Up to 50K	35.5	47.3	33.9	11.5
50K to 100K	41.6	36.1	44.0	38.9
100K and up	22.9	16.6	22.1	49.6
Education				
No secondary school	19.3	12.2	1.1	3.3
Secondary school	28.1	42.0	19.7	18.1
Diploma/trade	35.4	36.1	26.6	28.1
Bachelor’s degree	11.7	7.2	22.8	28.0
Higher degree	5.4	2.5	18.8	22.5
Area deprivation				
Least deprived	8.0	2.1	10.4	25.0
2	11.0	5.5	16.6	25.4
3	14.3	6.5	20.3	20.4
4	22.9	20.4	31.2	17.4
Most deprived	43.8	65.4	21.6	11.8

### Prevalence of racial discrimination

The prevalence of lifetime racial discrimination was higher than the prevalence of past year experiences. Lifetime experiences of verbal attacks were the most common experience of racial discrimination across all domains, and for all ethnic groups. In general, partners reported higher prevalence of lifetime and past year experiences of personal attacks than did mothers (see Table 2). Among mothers, Māori women reported the highest prevalence of experienced racial discrimination across all domains and time periods. Over a quarter (28.7 %) of Māori mothers reported ever experiencing a verbal attack because of their ethnicity, and almost 12 % reported experiencing verbal attacks in the past year. Prevalence of physical attacks was considerably lower, although experiencing any personal attack (verbal or physical) was nonetheless common among GUiNZ mothers (see Table 2).

**Table 2:** Prevalence of racial discrimination among mothers and partners in the Growing Up in New Zealand birth cohort study

	Māori		Pacific		Asian		European	
	Mother (*n* = 1,260) %	Partner (*n* = 653) %	Mother (*n* = 1,156) %	Partner (*n* = 583) %	Mother (*n* = 1,091) %	Partner (*n* = 638) %	Mother (*n* = 4,245) %	Partner (*n* = 3,000) %
Interpersonal attacks								
Physical attack, ever	4.2	9.2	2.9	7.4	2.4	5.3	2.5	6.9
Physical attack, past 12 months	1.4	^a^	1.3	2.2	^a^	2.4	0.5	1.0
Verbal attack, ever	28.7	32.6	17.7	25.3	22.8	32.0	17.0	21.6
Verbal attack, past 12 months	11.5	15.3	7.2	12.4	8.4	14.1	5.5	8.4
Any personal attack, ever	30.4	37.4	19.3	29.0	24.5	35.3	18.2	25.4
Any personal attack, past 12 months	12.0	16.2	8.0	13.4	9.1	15.9	5.8	9.0
Unfair treatment								
Health professional, ever	10.2	8.1	8.1	11.7	4.2	4.7	4.5	3.7
Health professional, past 12 months	5.9	4.9	4.6	4.8	2.6	2.8	2.3	1.8
Work, ever	7.7	9.8	6.9	14.8	13.3	15.6	3.9	4.5
Work, past 12 months	2.9	3.7	2.7	9.5	4.1	6.2	1.3	1.7
Housing, ever	11.7	8.3	8.3	8.8	5.3	6.9	2.6	1.9
Housing, past 12 months	4.5	2.8	4.1	3.1	2.0	2.4	1.1	0.6
Criminal justice system, ever	9.0	21.0	5.7	18.8	2.1	7.7	2.0	4.4
Criminal justice system, past 12 months	3.2	6.7	2.3	8.3	1.0	2.5	0.7	1.4
Banking system, ever	4.7	4.0	2.7	6.9	1.0	2.2	1.2	1.5
Banking system, past 12 months	1.7	2.1	1.3	4.3	^a^	^a^	0.4	0.8
Education system, ever	17.1	14.0	8.4	11.0	8.4	5.6	9.8	9.5
Education system, past 12 months	2.1	^a^	^a^	^a^	1.3	^a^	0.9	^a^
Any unfair treatment, ever	33.8	35.7	25.1	38.8	26.1	30.4	16.9	17.6
Any unfair treatment, past 12 months	13.6	14.1	12.1	21.0	10.0	12.2	5.2	4.9
One experience, ever	22.8	25.2	17.9	22.3	21.7	23.8	16.5	21.2
Two or more experiences, ever	23.4	27.2	15.5	26.9	15.5	22.0	10.4	12.5
One experience, past 12 months	12.3	15.5	12.0	16.4	11.0	16.3	6.5	8.9
Two or more experiences, past 12 months	7.9	8.1	5.2	11.2	4.4	6.3	2.5	2.8

^a^Cells have fewer than 10 cases. Exact numbers not reported to protect the anonymity of participants

Experiences of unfair treatment were most common within the health, housing, and education sectors. Lifetime and past year experiences of any unfair treatment were more common among Māori women, but were in general highly prevalent among all non-European mothers; 14 % of Māori, 12 % of Pacific, and 10 % of Asian mothers reported experiencing any unfair treatment in the 12 months before the antenatal interview (Table 2).

Among partners, Māori reported the highest prevalence of lifetime physical and verbal attacks (9 and 33 % respectively), and of any personal attacks ever (37 %), and in the past 12 months (16 %). Experiences of unfair treatment were most commonly reported by partners who identified as Pacific (see Table 2), although Asian partners reported the highest prevalence of unfair treatment in work place settings (16 % for lifetime experiences, and 6 % for past year experiences). Māori partners reported the highest prevalence of unfair treatment by the criminal justice and educational systems, with Pacific partners also experiencing a relatively high prevalence of unfair treatment in these same settings.

### Racial discrimination and mental health among mothers

Table 3 presents the associations between lifetime and past year experiences of racial discrimination, and the three measures of maternal mental health among Māori, Pacific, and Asian mothers. Analyses focusing on prenatal perceived stress showed an association between lifetime and past year experiences of verbal and any personal attacks, and increased levels of perceived stress in the 4 weeks prior to the antenatal interview. All lifetime experiences of unfair treatment were associated with increased perceived stress during pregnancy. These associations were particularly strong for lifetime experiences of unfair treatment in work settings, and in the banking sector. Past year experiences of unfair treatment by a health professional and by the banking sector were associated with increased prenatal stress (see Table 3).

**Table 3:** Associations between lifetime and past year experiences of racial discrimination and mental health among Māori, Pacific and Asian women (*n* = 3355)

	Prenatal perceived stress		Prenatal depression		Postnatal depression^a^	
	Ever	Past 12 months	Ever	Past 12 months	Ever	Past 12 months
	Coeff. (95 % C.I)	Coeff. (95 % C.I)	O.R. (95 % C.I)	O.R. (95 % C.I)	O.R. (95 % C.I)	O.R. (95 % C.I)
Personal attack						
Physical attack	1.27 (−0.18–2.72)	1.14 (−1.20–3.49)	1.86 (1.13–3.06)**	0.82 (0.32–2.09)	1.02 (0.52–1.99)	0.79 (0.25–2.51)
Verbal attack	1.68 (1.06–2.29)***	1.62 (0.74–2.49)***	1.58 (1.24–2.00)***	1.61 (1.17–2.21)***	1.28 (0.95–1.72)	0.96 (0.62–1.48)
Any personal attack	1.68 (1.08–2.28) ***	1.50 (0.65–2.35)***	1.62 (1.29–2.05)***	1.56 (1.44–2.14)**	1.27 (0.94–1.70)	0.95 (0.62–1.44)
Unfair treatment						
Health professional	1.42 (0.44–2.39)***	1.79 (0.51–3.07)**	1.51 (1.06–2.16)*	1.40 (0.88–2.24)	1.66 (1.08–2.55)*	1.31 (0.73–2.33)
Work	2.08 (1.20–2.97)***	1.23 (−0.23–2.69)	1.78 (1.29–2.46)***	1.74 (1.04–2.91)*	1.18 (0.78–1.80)	1.27 (0.66–2.45)
Housing	1.51 (0.58–2.44)***	2.27 (0.81–3.74)***	1.41 (1.00–1.99)*	2.10 (1.28–3.43)***	1.45 (0.95–2.22)	1.00 (0.50–1.99)
Criminal justice system	1.22 (0.05–2.38)*	1.25 (−0.56–3.07)	1.18 (0.76–1.82)	1.81 (0.98–3.33)	1.29 (0.74–2.20)	0.30 (0.09–1.00)
Banking system	2.55 (1.05–4.04)***	1.21 (−1.06–3.48)	1.36 (0.78–2.36)	1.52 (0.68–3.40)	1.28 (0.64–2.54)	1.17 (0.41–3.35)
Education system	0.98 (0.15–1.82)*	1.75 (−0.50–3.99)	1.42 (1.04–1.95)*	1.67 (0.74–3.75)	1.17 (0.79–1.74)	1.93 (0.71–5.22)
Any unfair treatment	1.83 (1.25–2.40) ***	1.76 (0.95–2.56)***	1.60 (1.29–1.99)***	1.63 (1.21–2.17)***	1.55 (1.17–2.04)***	1.29 (0.90–1.87)
One experience	1.08 (0.43–1.73) ***	1.64 (0.84–2.44)***	1.27 (0.98–1.65)	1.55 (1.15–2.08)***	1.49 (1.08–2.05)*	1.37 (0.95–1.97)
Two or more experiences	2.65 (1.95–3.35) ***	2.02 (0.91–3.12)***	1.98 (1.52–2.57)***	1.84 (1.24–2.72)***	1.51 (1.08–2.11)*	0.90 (0.52–1.57)

^a^Additionally adjusted for prenatal depression

*p < 0.05, **p < 0.01, ***p < 0.001; All models adjusted for maternal age, ethnicity, relationship status, equivalised household income, maternal education, and area deprivation

The patterns of the associations between lifetime and past year experiences of racial discrimination, and depression during pregnancy were similar to those found for perceived stress during pregnancy. However, we found an association between lifetime experiences of physical attacks and prenatal depression, whereby Māori, Pacific, and Asian women who had ever experienced a physical attack because of their ethnicity were 86 % more likely to suffer from prenatal depression than women who did not report experiencing a physical attack (O.R. = 1.86, 95 % C.I. = 1.13–3.06). Lifetime and past year experiences of unfair treatment were also associated with increased odds of reporting prenatal depression (see Table 3). Ever experiencing unfair treatment by a health professional, and in work, housing, and education settings were also associated with increased odds of reporting prenatal depression. We found strong associations between unfair treatment in work and housing settings in the past year, and increased odds ratios of reporting prenatal depression. These associations were particularly severe for discrimination in the housing sector; Māori, Pacific, and Asian women who reported unfair treatment in the housing sector were more than twice as likely to suffer from depressive symptoms during pregnancy than those who did not report experiencing unfair treatment in the housing sector during the past year (O.R. = 2.10, 95 % C.I. = 1.28–3.43).

We found a dose-response relationship between the number of types of lifetime and past year experiences of racial discrimination and the two measures of prenatal mental health, as the size of the association between experiences of racial discrimination and poor mental health strengthened with each increase in level of exposure to racial discrimination (see Table 3).

Postnatal depression was detrimentally associated with lifetime experiences of unfair treatment by a health professional (O.R. = 1.66, 95 % C.I. = 1.08–2.55). The summary measure of lifetime unfair treatment, and the levels of exposure to lifetime racial discrimination were also associated with increased odds of suffering from postnatal depression (see Table 3).

### Mother’s and partner’s experiences of racial discrimination, and mother’s mental health

Tables 4 and 5 present results for the individual and joint contribution of mother’s and partner’s experiences of lifetime and past year racial discrimination to women’s prenatal and postnatal mental health. Mothers in a partnership where both parents (mothers and their partners) had experienced a racist personal attack in their lifetime had much higher levels of prenatal perceived stress, and higher odds ratios of prenatal and postnatal depression, than women who neither themselves, nor their partners, had experienced a personal attack (see Table 4). Mothers who had experienced a personal attack in their lifetime, but whose partners had not reported these experiences, still reported higher levels of prenatal perceived stress than women who themselves, and their partners, reported never experiencing racial discrimination.

**Table 4:** Associations between mother’s and partner’s lifetime experiences of racial discrimination and maternal prenatal and postnatal mental health (*n* = 4118)

	Prenatal perceived stress	Prenatal depression	Postnatal depression^a^
	Coeff. (95 % C.I)	O.R. (95 % C.I)	O.R. (95 % C.I)
Nobody experienced personal attack	Reference	Reference	Reference
Mother experienced personal attack	1.32 (0.69–1.95)***	1.44 (1.06–1.95)*	1.43 (0.97–2.06)
Partner experienced personal attack	0.41 (−0.09–0.92)	1.32 (1.02–1.69)*	1.16 (0.84–1.60)
Both experienced personal attack	2.09 (1.28–2.90)***	1.85 (1.30–2.63)***	1.66 (1.09–2.54)*
Nobody treated unfairly	Reference	Reference	Reference
Mother treated unfairly	1.60 (0.97–2.24)***	1.85 (1.39–2.46)***	1.71 (1.22–2.40)***
Partner treated unfairly	0.40 (−0.15–0.95)	1.42 (1.08–1.86)**	1.12 (0.80–1.57)
Both treated unfairly	2.28 (1.42–3.14)***	1.88 (1.31–2.71)***	0.92 (0.55–1.53)

^a^Additionally adjusted for prenatal depression

*p < 0.05, **p < 0.01, ***p < 0.001; Models adjust for maternal age, equivalised household income, maternal education, mother’s and partner’s ethnicity, and area deprivation

**Table 5:** Associations between mother’s and partner’s past year experiences of racial discrimination and maternal prenatal and postnatal mental health (*n* = 4118)

	Prenatal perceived stress	Prenatal depression	Postnatal depression^a^
	Coeff. (95 % C.I)	O.R. (95 % C.I)	O.R. (95 % C.I)
Nobody experienced personal attack	Reference	Reference	Reference
Mother experienced personal attack	1.05 (0.12–1.98)*	1.47 (0.98–2.20)	1.48 (0.92–2.38)
Partner experienced personal attack	0.28 (−0.40–0.96)	1.20 (0.87–1.65)	1.03 (0.69–1.56)
Both experienced personal attack	1.27 (−0.49–3.03)	0.94 (0.41–2.15)	1.72 (0.72–4.07)
Nobody treated unfairly	Reference	Reference	Reference
Mother treated unfairly	2.58 (1.66–3.50)***	2.24 (1.56–3.22)***	1.51 (0.97–2.35)
Partner treated unfairly	0.43 (−0.35–1.21)	1.28 (0.90–1.83)	0.63 (0.37–1.04)
Both treated unfairly	3.10 (1.28–4.92)***	2.01 (1.00–4.06)*	1.23 (0.50–3.04)

^a^Additionally adjusted for prenatal depression

*p < 0.05, ***p < 0.001; Models adjust for maternal age, equivalised household income, maternal education, mother’s and partner’s ethnicity, and area deprivation

Women who were in partnerships where only they had experienced unfair treatment reported increased levels of perceived prenatal stress, prenatal depression, and postnatal depression (Table 4). Prenatal stress was much higher for mothers in partnerships where both themselves and their partner had been treated unfairly in their lifetime, as compared to mothers who neither themselves, nor their partners had experienced any unfair treatment. Mothers who were in couples where both partners had been treated unfairly were over 88 % more likely to report prenatal depression than mothers who were in couples where nobody had reported unfair treatment (Table 4).

Lifetime experiences of personal attacks and unfair treatment reported by both the mother and the partner, and lifetime experience of unfair treatment by the mother only, were also associated with increased odds of reporting postnatal depression (see Table 4).

Mothers who did not report lifetime experiences of personal attacks or unfair treatment, but whose partners reported these experiences, had higher odds ratios of prenatal depression as compared to women in a partnership where nobody had experienced any racial discrimination. We did not find any associations between partner’s experiences of racial discrimination and prenatal stress or postnatal depression (Table 4).

Maternal reports of unfair treatment in the past year, and reports that both the mother and the partner had experienced unfair treatment in the past year, were associated with increased levels of prenatal perceived stress and prenatal depression (see Table 5). Maternal reports of personal attacks were also associated with increased levels of prenatal stress. None of the measures of past year experiences of personal attacks (by the mother, the partner, or both) were associated with postnatal depression (see Table 5).

## Discussion

This study set out to examine, in an ethnically diverse longitudinal birth cohort in New Zealand, the contribution of lifetime and past year experiences of racial discrimination to prenatal and postnatal depression and perceived stress. The first aim of the study was to document the prevalence of lifetime and past year experiences of racial discrimination among pregnant women and their partners. The overall patterns and prevalence of racial discrimination described by the parents of the Growing Up in New Zealand cohort are similar to those documented in other studies or racial discrimination using nationally representative data from New Zealand [40]. Previous research in New Zealand has also found similar patterns regarding discrimination, and the highest level of discrimination for all ethnicities was reported in the employment setting. Overall, 4 % (approximately 143,000 New Zealanders) reported that they had been discriminated against while at work or while applying for a job. It is particularly recognised in the work place setting that racial discrimination is not only associated with ethnicity but also with place of birth - with migrant more commonly reporting workplace discrimination than non-migrants, and increased reporting of racial discrimination increases with time spent in country [55]. Language and accent have also been described as predictors of the experience of racial discrimination [56]. In New Zealand, people who hold a formal educational qualification have also been found to more likely to report racial discrimination in the employment setting. The importance of racial discrimination in the health care setting for the indigenous Māori population has been described and there is growing evidence of the contribution of racism to ethnic inequities in health in New Zealand [41]. Measures of racial discrimination have been collected in the New Zealand Health Survey in 2002/3 and 2006/7, and Maori reported the highest prevalence of discrimination because of ethnicity at both time points. In 2006/7 Māori reported the highest proportion who had experienced racial discrimination in the health sector [40]. In New Zealand the context of racial discrimination towards Māori involves a history of colonisation, dispossession, marginalisation and unequal power relationships in society and societal structures [57]. The overlapping societal, institutional, interpersonal and internalised levels of racism [4] in New Zealand are commonly perpetuated by social norms, the media, and institutions [58].

The second aim of the present study examined the burden of lifetime and past year experiences of racial discrimination on prenatal and postnatal mental health among Māori, Pacific, and Asian women. Our findings show strong associations between lifetime and past year experiences of interpersonal attacks and unfair treatment on prenatal stress and depression. In terms of postnatal depression, we found that Māori, Pacific, and Asian women who had experienced unfair treatment by a health professional in their lifetime were 88 % more likely to suffer from postnatal depression, than women who did not report these experiences. Across both prenatal mental health outcomes and time frames, we found a dose-response association between experiences of personal attacks, and experiences of unfair treatment, so that the association between racial discrimination and prenatal mental distress strengthened as the number of domains of racial discrimination experienced increased.

Among experiences of unfair treatment reported to have occurred in the period around conception and delivery (i.e., in the past 12 months), experiencing unfair treatment by a health professional, in the housing sector, and in work settings had the strongest associations with increased prenatal perceived stress and prenatal depression. The increased relevance of racial discrimination in health care settings on mental health has been previously documented in a study among Aboriginal people in Australia, where racism in health settings was found to have a more negative association with mental health than racism in other settings [59]. Experiences of racial discrimination in health care, housing, and employment settings not only have severe direct consequences for the mother’s health and the children’s development via prenatal depression and increased stress during pregnancy, as we describe below, but also have implications for the basic human needs of a healthy child. These particular domains – healthcare, housing, employment – are key in ensuring that new-borns live in good quality housing, receive appropriate healthcare, and grow up in a household with sufficient economic means to guarantee access to a healthy diet, a safe and stimulating environment, and other factors that are crucial to achieve children’s full potential [60]. For example, the quality of housing, and the difficulty in accessing adequate and affordable housing, is recognised to contribute to inequitable outcomes for children in New Zealand [61]. Racial discrimination experienced in these crucial domains has therefore long-term repercussions for the development of the child, independently to the consequences of maternal stress and depression.

In terms of the association between racial discrimination and maternal mental health, excess maternal stress at critical periods of the foetus’ development is associated with a wide range of risk factors for psychopathology as children develop [62], and maternal stress that continues in postnatal stages also has been found to have a role in children’s development [62]. Depression during pregnancy is associated with obstetric complications and preterm delivery [63, 64], and with short and long-term consequences on the child’s development [65–72]. After birth, postnatal depression is associated with children’s cognitive and emotional development [60], family homelessness [73], and children’s delayed language and behaviour problems [74]. Although findings from Aim 2 show the severe consequences of maternal experiences of racial discrimination in perpetuating health and social inequalities, they focus only on the mother’s experiences, and do not capture how racial discrimination experienced by other adults in her social network is associated with maternal mental health, and with the subsequent health and development of the new-born. In Aim 3, we widened the circle of exposure to racial discrimination around the child, and examined, among women whose partners had completed an antenatal interview, the individual and joint contribution of mother’s and partner’s experiences of lifetime and past year personal attacks and unfair treatment to prenatal and postnatal maternal mental health. Across the three mental health outcomes, we found evidence for a cumulative effect of lifetime experiences of personal attacks on poor maternal mental health if both the mother and the partner had experienced a racist attack. Across all outcomes and time points, associations between experiences of racial discrimination and poor mental health occurred if at least the mother reported these experiences. In most cases, these associations strengthened if the partner had also suffered from racial discrimination. We found that only in the case of prenatal depression, the partner’s sole experience of experiencing racial discrimination was sufficient to increase the mother’s likelihood of developing prenatal depression. Other work has shown that family experiences of racial discrimination are independently associated with maternal psychological distress [21], and in the present study we find additional evidence to support this. To the cumulative effect of partner’s experiences of racial discrimination on maternal mental health, we should also add the additional burden to social inequalities from the analyses from Aim 1. We found that 21 % of Māori partners and 18 % of Pacific partners had been treated unfairly by criminal justice system in their lifetimes; that 10 % of Māori, 15 % of Pacific, and 16 % of Asian partners had ever been treated unfairly in work settings; and that 14 % of Māori, 11 % of Pacific, and 6 % of Asian partners had ever been treated unfairly by the education system. These are the highest prevalences of unfair treatment across key social indicators, but experiences of unfair treatment were reported across the other domains, all of which lead to social and health inequalities of the parents, and of children. By considering how racial discrimination – through personal attacks and unfair treatment – harms the mental health of the mother, and the chances for a good job, good quality housing, adequate health care, equal treatment from the criminal and banking systems, and a good education, of the mother, the partner, and ultimately, the child, we have aimed to show the extensive and long-term harm on New Zealand Māori, Pacific, and Asian families.

Limitations of this study ought to be acknowledged. First, although we had longitudinal data to assess the association between lifetime and past-year exposure to racial discrimination and postnatal depression, for the prenatal outcomes we only had access to cross-sectional data. This precludes us from assessing direction of causality, although evidence now supports that exposure to racial discrimination predates poor health [75–80]. Another limitation that arises from our study design is that women who did not have a partner at the time of enrolment in the Growing Up in New Zealand study may have had partners who have experienced racial discrimination in the past, which may have affected their mental health, and which is not captured here. It is also possible that women may have had partners who did not enrol in the study, and who may have experienced racial discrimination. This means that we may have underestimated the association between partner’s experience of racial discrimination, and women’s prenatal and postnatal mental health. Although we were able to assess a dose-response relationship across domains, we would have liked to also examine cumulative exposure across time. Unfortunately we were not able to do so with the data from the Growing Up in New Zealand cohort for this study, but one could expect that experiences of unfair treatment in domains such as healthcare occur repeatedly, especially in life course events that require frequent medical care, as are pregnancy and early child development. Vigilance and anticipatory stress of future experiences of racial discrimination have been associated with poor health outcomes [81], and in this case, vigilance and anticipatory stress may result from fear that the mothers themselves, and their children, might be the victims of racial discrimination in a health care setting. In future waves of the Growing Up in New Zealand study, ongoing measurement of the experience for families within areas such as the health, social, and educational sectors will ensure that researchers are able to examine whether exposure to unfair treatment in these domains occurs continuously through time, and the association between repeated victimisation and the health of the mother and the child.

## Conclusion

Despite the study’s limitations, our findings show the detrimental associations between lifetime and past-year experiences of maternal and partner personal attacks and unfair treatment, and maternal prenatal perceived stress, prenatal depression, and postnatal mental health. Using a diverse longitudinal birth cohort from New Zealand, our study shows the high prevalence of experiences of interpersonal racism among women and their partners, and the harm that these experiences have for the mental health of the mother. Interventions designed to treat postnatal depression and the mother-infant relationship show improvements in child cognitive development [71]. These are important and promising findings, but treating depression among women who have experienced racial discrimination, although an important public health intervention, addresses the consequence, and not the root of the problem. As our results show, the high prevalence of racial discrimination in experiences of personal attack and unfair treatment – of both mothers and partners – requires that focus is placed on racism and racial discrimination, the fundamental causes that lead to poor maternal and children’s health, and perpetuate ethnic social and health inequalities across families and generations.

## Abbreviations

GUiNZ: Growing up in New Zealand
